# General anesthesia and positive pressure ventilation suppress left and right ventricular myocardial shortening in patients without myocardial disease – a strain echocardiography study

**DOI:** 10.1186/s12947-019-0165-z

**Published:** 2019-08-10

**Authors:** Keti Dalla, Odd Bech-Hanssen, Sven-Erik Ricksten

**Affiliations:** 1 Department of Anaesthesiology and Intensive Care Medicine, Sahlgrenska Academy, University of Gothenburg, Sahlgrenska University Hospital, Gothenburg, Sweden; 2 Department of Clinical Physiology, Sahlgrenska Academy, University of Gothenburg, Sahlgrenska University Hospital, Gothenburg, Sweden

**Keywords:** Ventricular function, Left ventricular elastance, Strain echocardiography, Anaesthesia, Positive pressure ventilation

## Abstract

**Background:**

Myocardial deformation imaging using speckle-tracking echocardiography to assess global longitudinal strain (GLS) is today considered a more sensitive measure of left ventricular (LV) systolic function than ejection fraction. General anesthesia and positive pressure ventilation (PPV) are known to change the right ventricular (RV) and LV loading conditions. However, little is known about the effects of anesthesia and PPV on RV free wall and LV GLS. We studied the influence of general anesthesia and PPV on RV and LV longitudinal strain in patients without myocardial disease.

**Methods:**

Twenty-one patients scheduled for non-cardiac surgery were included. The baseline examination was performed on the un-premedicated patients within 60 min of anesthesia. The second examination was performed 10–15 min after induction of anesthesia (propofol, remifentanil), intubation and start of PPV. The examinations included apical four-, two- and three-chamber projections, mitral and aortic Doppler flow velocities and tissue Doppler velocities of tricuspid and mitral annulus. LV end-systolic elastance (Ees) and aortic elastance were determined (Ea).

**Results:**

General anesthesia and PPV reduced the mean arterial blood pressure (− 29%, *p* <  0.0019), stroke volume index (− 13%, p <  0.001) and cardiac index (− 23%, p <  0.001). RV end-diastolic area index and LV end-diastolic volume index decreased significantly, while systemic vascular resistance was not significantly affected. Ees decreased significantly with the induction of anaesthesia (− 23%, *p* = 0.002), while there was a trend for a decrease in Ea (*p* = 0.053). The ventriculo-arterial coupling, Ea/Ees, was not significantly affected by the anesthetics and PPV. The LV GLS decreased from − 19.1 ± 2.3% to − 17.3 ± 2.9% (*p* <  0.001) and RV free wall strain decreased from − 26.5 ± 3.9% to − 24.1 ± 4.2% (*p* = 0.001). One patient (5%) had at baseline a LV GLS > − 16% compared with 6 patients (28%) during general anesthesia and PPV. Three patients (14%) had a RV free wall strain > − 24% compared to 8 patients (38%) during general anesthesia and PPV.

**Conclusions:**

General anesthesia and PPV reduces systolic LV and RV function to levels considered indicating dysfunction in a substantial proportion of patients without myocardial disease.

## Background

Conventional two-dimensional echocardiography is the method of choice for the evaluation of left (LV) and right (RV) ventricular global and regional myocardial function in patients undergoing surgery and in the critically ill patient. Two-dimensional speckle tracking echocardiography is a relatively new method, which is increasingly used to detect LV and RV dysfunction [1]. Speckle tracking echocardiography is an angle-independent method, which quantifies systolic function of LV and RV by the assessment of systolic myocardial deformation, strain. Strain is a negative dimension-less variable, describing percentage changes in myocardial segment length [1–3]. The most frequently used strain variable, global longitudinal strain (GLS), measures the contractile function of longitudinally oriented subendocardial myocardial fibers, which are more sensitive to ischemia and increased wall stress [4]. Longitudinal RV free wall strain is also a robust measure of RV systolic function. Intra-observer and inter-observer reproducibility of myocardial strain measurements is good and in many cases superior to conventional echocardiographic measurements [5].

Experimental studies have shown that myocardial strain is a load-dependent index [6–9]. Data, however, in conscious patients are divergent [10–13]. Changes in cardiac loading condition, such as hypotension, are commonly seen in patients undergoing total intravenous anaesthesia for surgical procedures. Propofol is a commonly used intravenous anaesthetic, which is usually combined with an opioid (e.g. remifentanil). There are, to our knowledge, no studies investigating the effects of propofol/remifentanil on myocardial function assessed by LV GLS or RV free wall strain.

Mechanical ventilation with the application of positive end-expiratory pressure (PEEP) increases intrathoracic pressure, which will affect venous return and cardiac output [14] and thus has the potential to affect cardiac loading conditions. There are, however, no data on the effects of the transition from spontaneous breathing to positive pressure breathing on myocardial strain of the left and right ventricle in patients with normal cardiac function.

In the ICU, myocardial dysfunction occurs frequently and speckle tracking echocardiography has the ability to detect impaired LV systolic function not appreciated by conventional echocardiography [15, 16] . Patients admitted to ICU often require sedation and positive-pressure ventilation which may potentially change the LV and RV loading conditions and myocardial contractility. However, little is known about the combined effects of anesthesia/sedation and positive pressure ventilation, per se, on myocardial strain. The aim of the present study was therefore to investigate the influence of general anaesthesia and positive pressure ventilation on myocardial longitudinal strain in patients without myocardial disease.

## Methods

The study was approved by the Regional Ethical Review Board in Gothenburg (www.epn.se) (protocol no. 477–17, approved: July 27th 2017). Written informed consent was obtained from all patients.

### Study population

Patients scheduled for non-cardiac surgery were included in this study. The inclusion criteria were: a) Low-risk (ASA I-II) elective surgery, b) surgery planned to be performed under total intravenous anaesthesia and positive pressure ventilation and c) informed consent was obtained. Exclusion criteria were: a) history or clinical or laboratory signs of cardiac, pulmonary or systemic disease, b) any cardiac or antihypertensive medication, c) abnormal ECG d) age < 18 year and e) a body mass index ≥30 kg m^− 2^.

### Echocardiography

Two transthoracic 2D echocardiographic examinations were performed with a 5-MH transducer (Vivid E9, General Electric Medical System, Horten, Norway one before and one directly after the induction of anaesthesia and initiation of IPPV. The examinations included apical four-, two- and three-chamber projections, mitral and aortic Doppler flow velocities. Standard measurements of LV systolic function included LV volumes (indexed to body surface area, BSA) left ventricular ejection fraction (LVEF) by the modified Simpson’s rule, time velocity integral in the LV outflow tract (TVI-LVOT) and stroke volume (SV) (= π x LVOT radius^2^ x TVI-LVOT). Stroke volume index (SVI) was calculated as SV/ BSA. Mitral and aortic Doppler flow profiles were recorded for measurements of LV isovolumetric relaxation time, maximum flow velocity during LV early (E-max) and late (A-max) diastolic filling. RV systolic function was assessed by using tricuspid annular plane systolic excursion (TAPSE) by M-mode and tricuspid lateral annulus tissue Doppler systolic velocity. RV end-diastolic and end-systolic area were measured (indexed to BSA) and RV fractional area change (%) were calculated.

### Haemodynamic measurements

Systolic (SAP) and diastolic (DAP) arterial blood pressure were measured non-invasively and intermittently at 5 min interval, using an occluding upper-arm cuff of suitable size in the supine position and mean arterial pressure (MAP) were calculated. Heart rate and arterial blood pressure were recorded just before and during the echocardiographic examination. Systemic vascular resistance index was calculated according to standard formula (MAP/cardiac output)× 80 /BSA.

Effective arterial elastance (Ea) was measured as 0.9 x SAP / SV. Ea incorporates all elements of total LV afterload, including vascular resistance, arterial compliance and characteristic impedance.

The LV end-systolic elastance Ees, a load-independent measure of myocardial contractility, was calculated according to single-beat method described by Chen et al. [17] using the following formulas:

Ees(sb) = [DBP - (ENd(est) × SBP × 0.9)] / [SV × ENd(est)], where.

Ees(sb) is the single-beat LV end-systolic elastance. ENd(est) is the noninvasively estimated normalized elastance at the onset of ejection and is calculated as:

ENd(est) = 0.0275–0.165 × EF × (DAP/SAP × 0.9) + 0.515 × ENd (avg), where.

EF is the LV ejection fraction and ENd (avg) is calculated as:

ENd (avg) = ∑ a_i_ x tNd^i^, where ai are (0.35695, − 7.2266, 74.249, − 307.39, 684.54,

− 856.92, 571.95, − 159.1) for i = 0 to 7, respectively and tNd is the ratio of pre-ejection period to total systolic period.

### Strain echocardiography

Strain measurements were performed off-line in the four-chamber, long axis- and two-chamber views. All off-line analyses were performed by an investigator experienced in speckle tracking analysis using the EchoPAC workstation version 201(GE Medical Systems, Milwaukee, Wisconsin, USA). From the strain analysis, we calculated the longitudinal strain of the free RV wall and the global longitudinal strain (GLS) for the LV. Myocardial strain (S) is presented as fractional change (%) in length between two time points, end-diastole (L_0_) and end-systole (L) and calculated as: (L – L_0_)/L_0_ × 100. Negative values of strain indicate myocardial shortening. Impaired LV GLS and RV free wall strain was defined as > − 16% [18] and > − 24% [19] respectively.

### Experimental protocol

The first (baseline) transthoracic echocardiography (TTE) was performed after the arrival in the preoperative area within 60 min before induction of anesthesia, with the patient awake, un-premedicated and in a partial left lateral position. Ten to fifteen minutes after induction of anaesthesia, intubation and start of intermittent positive pressure ventilation (IPPV), the second echocardiographic examination was performed also in a partial left lateral position by the same investigator. General anaesthesia was induced and maintained by infusion of propofol and remifentanil. Rocuronium 0.6 mg kg^− 1^ was administered before the tracheal intubation. PPV to normocapnia (end-tidal carbon dioxide 4.5–5 kPa) was commenced with ventilator settings at the discretion of the attending anaesthesiologist. Hypotension, defined as a MAP < 60 mmHg was treated with i.v. bolus doses of 50 mg phenylephrine or 5 mg ephedrine.

### Statistics

The intra-observer agreement of RV free wall strain and LV global longitudinal strain were assessed by the coefficients of variation for paired observations of RV and LV strain from the measurements of the first (baseline) examination. Our data were normally distributed and expressed as mean ± SD. To detect a difference in LV GLS of 1.5% units, 17 patients were needed to be included at a standard deviation of the mean differences of paired measurements of 2. Paired t-test was used to compare the means before and after induction of anaesthesia. A probability level (*p*-value) of less than 0.05 was considered to indicate statistical significance. Statistical analysis was performed using SPSS for Mac version 21.

## Results

Twenty-one patients were included in the study, 11 male and 10 females with a mean age of 47 ± 15 years (Table 1). Data on the doses of propofol and remifentanil and the ventilatory settings are shown in Table 1. The bispectral index (BIS) was used to determine the anaesthetic depth [20]. The BIS level was 39 ± 9 after induction of anaesthesia. A BIS value between 40 and 60 is considered to be an appropriate level for general anaesthesia.

**Table 1:** Patient characteristics, anaesthetics and mode of ventilation

	*n* = 21
Age (years)	47 ± 15
Female gender (%)	47
Body surface area (m^2^)	1.8 ± 0.3
Propofol (mg/kg/h)	8.6 ± 2.9
Remifentanil (μg/kg/min)	0.11 ± 0.04
Bispectral index (%	39 ± 9
Respiratory rate (breaths/min)	13 ± 2
Tidal volume (ml)	422 ± 73
Inspired fraction of oxygen (%)	32 ± 3
Positive end-expiratory pressure (mmHg)	5 ± 3

Data are presented as means ± SD

### Hemodynamic variables

The induction of total intravenous anaesthesia combined with positive pressure ventilation was associated with a significant reduction of mean, systolic and diastolic arterial blood pressure (*p* <  0.001) (Table 2). Two patients needed one bolus dose of ephedrine and one patient received one bolus dose of phenylephrine to maintain mean arterial pressure > 60 mmHg. The fall in arterial blood pressure was accompanied by a decrease in stroke volume index (− 13%, p <  0.001), cardiac index (− 23%, p <  0.001) and heart rate (− 8%, *p* = 0.038), while systemic vascular resistance index was not affected. Ees decreased significantly with the induction of anaesthesia (− 23%, *p* = 0.002), while there was a trend for a decrease in Ea (*p* = 0.053). The ventriculao-arterial coupling, Ea/Ees, were not significantly affected by the anesthetics and PPV (*p* = 0.102).

**Table 2:** Haemodynamic data

	Awake patient	Anaesthesia + PPV	p-value
Mean arterial pressure (mmHg)	91 ± 14	65 ± 8	< 0.001
Systolic arterial pressure (mmHg)	124 ± 21	93 ± 10	< 0.001
Diastolic arterial pressure (mmHg)	76 ± 13	54 ± 8	< 0.001
Stroke volume index(ml/m^2^)	37 ± 11	32 ± 9	< 0.001
Cardiac output (l/min)	4.6 ± 1.1	3.6 ± 0.9	< 0.001
Cardiac index (l/min/m^2^)	2.6 ± 0.7	2.0 ± 0.7	< 0.001
Heart rate (beats/min)	72 ± 16	66 ± 14	0.038
Left ventricular elastance (mmHg/ml) (Ees)	2.6 ± 0.7	2.0 ± 0.7	0.002
Arterial elastance (mmHg/ml) (Ea)	1.8 ± 0.4	1.6 ± 0.3	0.053
Ea/Ees	0.71 ± 0.12	0.94 ± 0.65	0.102
SVRI (dynes x sec/cm^5^/m^2^)	906 ± 189	879 ± 257	0.565

PPV; positive pressure ventilation, SVRI: systemic vascular resistance index

### Echocardiographic variables (Table 3, Figs. 1, 2, 3 and 4)

#### Left ventricle

After induction of anaesthesia combined with positive pressure ventilation a decrease was observed in LV GLS (− 10%, *p* <  0.001), LV end-diastolic volume index (− 17%, *p* = 0.012) and TVI-LVOT (− 7%, *p* <  0.001), while LVEF or LV end-systolic volume index was not affected. A decrease was observed in E-max (− 17%, *p* <  0.001) and A-max (− 27%, p <  0.001), while LV isovolumic relaxation time was not affected after induction anaesthesia combined with positive pressure ventilation.

**Table 3:** Echocardiographic data

	Awake patient	Anaesthesia + PPV	p-value
Left ventricular global longitudinal strain (%)	−19.1 ± 2.3	−17.3 ± 2.9	< 0.001
Left ventricular end-diastolic volume index (ml/m^2^)	57 ± 18	47 ± 18	0.012
Left ventricular end-systolic volume index (ml/m^2^)	23 ± 8	20 ± 9	0.084
Time velocity integral of the LV outflow tract (cm)	18.6 ± 3.0	17.3 ± 2.9	< 0.001
Left ventricular ejection fraction (%)	59 ± 8	56 ± 10	0.130
E-max (cm/sec)	69 ± 15	57 ± 13	< 0.001
A-max (cm/sec)	59 ± 19	43 ± 14	0.001
Left ventricular isovolumic relaxation time (ms)	69 ± 17	67 ± 21	0.781
Right ventricular free wall strain (%)	−26.8 ± 3.9	−24.1 ± 4.2	0.001
Tricuspid annular peak systolic velocity (cm/sec)	11.4 ± 3.0	9.2 ± 2.4	< 0.001
Tricuspid annular plane tissue doppler systolic excursion (mm)	2.4 ± 0.5	1.9 ± 0.4	< 0.001
Right ventricular end-diastolic area index (cm^2^/m^2^)	13 ± 3	12 ± 2	0.007
Right ventricular end-systolic area index (cm^2^/m^2^)	7 ± 2	7 ± 1	0.782
Right ventricular fractional area change (%)	46 ± 7	39 ± 7	0.013

PPV; positive pressure ventilation, E-max; maximum flow velocity during early LV diastolic filling, A-max; maximum flow velocity during late diastolic LV filling

**Fig. 1:**
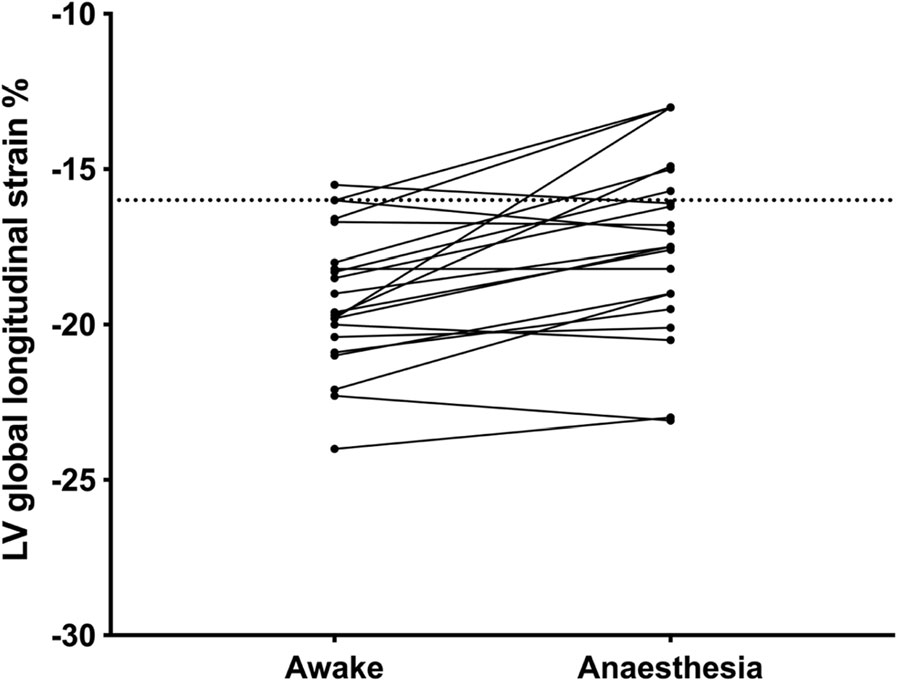
Shows the effects of anaesthesia and positive pressure ventilation (PPV) on left ventricular (LV) global longitudinal strain (GLS). In the majority of patients, LV GLS was impaired after induction of anaesthesia

**Fig. 2:**
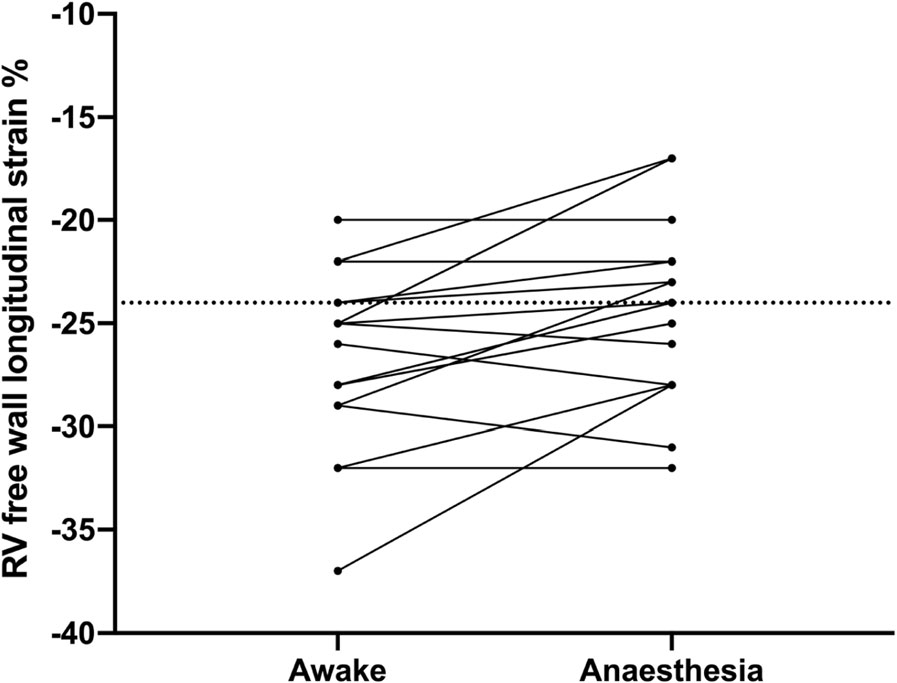
Shows the effects of anaesthesia and positive pressure ventilation (PPV) on right ventricular (RV) free wall strain. In the majority of patients, RV free wall strain was impaired after induction of anaesthesia

**Fig. 3:**
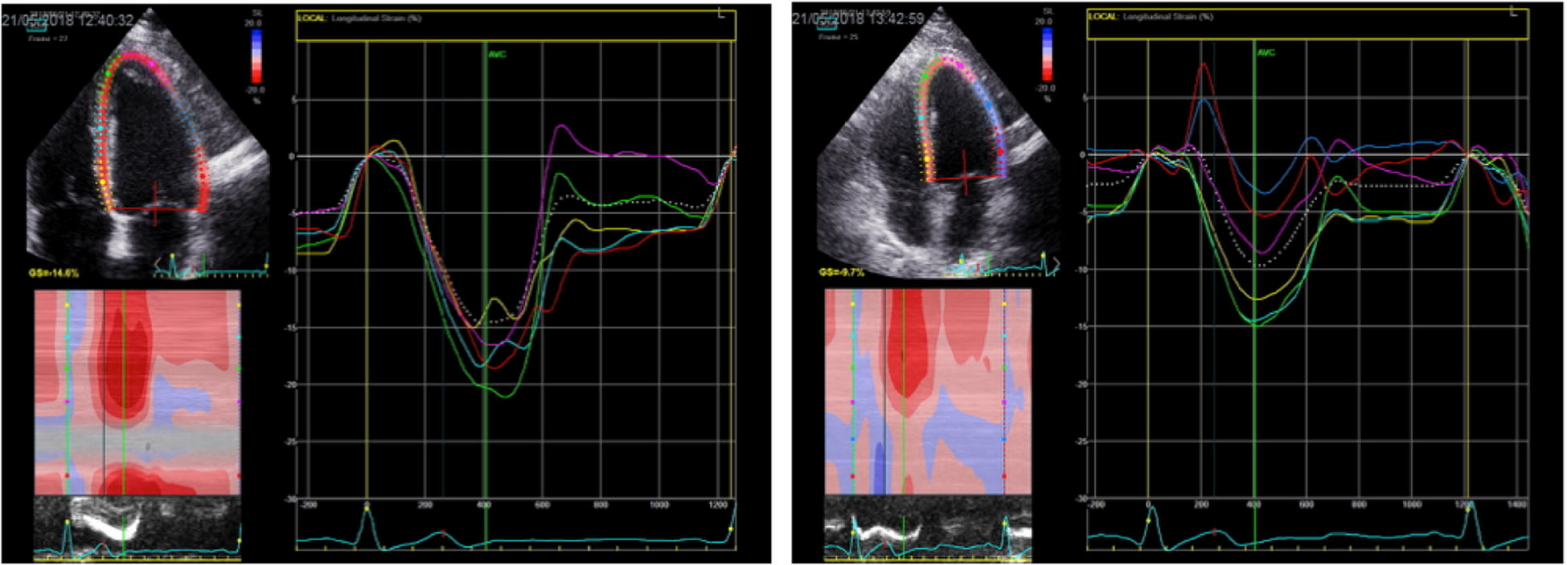
Shows 4-chamber recordings of left ventricular global longitudinal strain before and after anaesthesia and positive pressure ventilation (PPV)

**Fig. 4:**
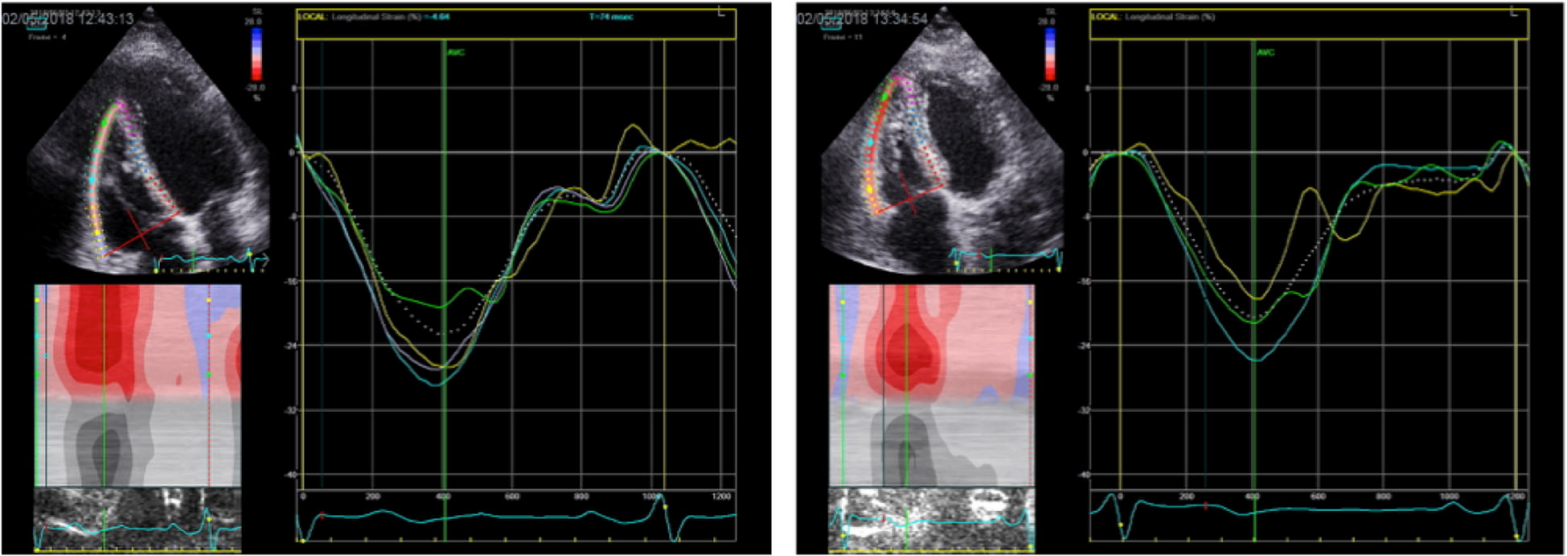
Shows 4-chamber recordings of right ventricular free wall strain before and after anaesthesia and positive pressure ventilation (PPV)

#### Right ventricle

After induction of anaesthesia combined with positive pressure ventilation a decrease was observed in RV free wall strain (− 10%, *p* = 0.001), tricuspid annular peak systolic velocity (− 19%, p <  0.001), tricuspid annular plane systolic excursion (− 21%, p <  0.001), RV fractional area change (− 15%, *p* = 0.013) and RV end-diastolic area index (− 7%, *p* = 0.007). RV end-systolic area index was not affected by anaesthesia with PPV.

One patient (5%) had an impaired GLS at baseline (GLS < − 16%) compared with six patients (20%) during general anesthesia and positive pressure ventilation (Fig. 1). Three patients (14%) had impaired RV free wall strain at baseline (> − 24%), while eight (38%) had impaired RV free wall strain during general anesthesia and positive pressure ventilation (Fig. 2).

The intra-observer coefficient of variation for repeated measurements of RV free wall and LV global longitudinal strain were 11.9 and 8.8% respectively.

## Discussion

In the present study we evaluated the influence of general anaesthesia and positive pressure ventilation (PPV) on myocardial systolic function evaluated by LV global longitudinal strain (GLS) and RV free wall strain. The main findings of the study were that general anaesthesia plus PPV induced a significant reduction of LV GLS and RV free wall strain and that in some patients the reduction of GLS and RV free wall strain reached values considered to indicate LV or RV dysfunction. This could be explained by changes in myocardial loading conditions and myocardial contractility caused by the intravenous anaesthetics combined with PPV. Thus, in the evaluation of myocardial function in anaesthetised/sedated mechanically ventilated patients during surgery, or in the critical care unit, systolic function may be underestimated by strain echocardiography.

In the present study, anaesthesia and PPV caused a decrease in preload, as assessed by the fall in RV end-diastolic area index (RVEDAI) and LV end-diastolic volume index (LVEDVI). The reduction in preload is also supported by a decrease in maximum flow velocity during LV early (E-max) and late (A-max) diastolic filling. The fall in LVEDVI may to some extent also explain the decrease in stroke volume and cardiac output. The decrease in cardiac output explained the fall in MAP, as systemic vascular resistance was not affected by anaesthesia and PPV. Previous experimental studies have shown that strain is a preload-dependent index [6–9]. However, the preload-dependency of strain has been investigated in conscious patients with divergent results [10–13]. Abali et al. showed that 500 ml of blood donation from healthy volunteers decreased LV strain, measured by tissue Doppler [11]. Mendes et al. evaluated the effects of haemodialysis on systolic and diastolic function in patients with end-stage renal disease using STE and tissue Doppler imaging. They found that the preload reduction had no effects on strain [12]. Burns et al. induced a preload reduction on patients by nitroglycerin, which caused a substantial fall in LV end-diastolic filling pressure and volume, as well as, arterial blood pressure [13]. This preload reduction increased LV strain. The same authors also increased preload by saline fluid loading in this patient group and found that volume loading did not affect LV strain. Finally, Andersen et al. could not show a significant influence on LV systolic strain by load alterations using passive leg elevation or administration of nitroglycerin sublingually in healthy volunteers [10]. This could be explained by the fact that changes in preload (nitroglycerin, hypovolemia, volume loading) or afterload (nitroglycerin, phenylephrine) will induce arterial- and cardiac baroreceptor-mediated reflex counterregulatory changes in cardiac sympathetic activity, which will increase/decrease cardiac contractility depending on the haemodynamic stimulation and thereby affect LV strain.

In a recent clinical strain-echocardiograhic study, Fredholm et al. evaluated the load-dependence of myocardial deformation variables in mechanically ventilated propofol-sedated postcardiac surgery patients. Propofol is known to induce a considerable attenuation of the baroreflex sensitivity [21] and therefore could the confounding effects of baroreceptor-mediated changes in cardiac performance to a considerable extent be eliminated [22]. In that study it was shown that myocardial strain is particularly sensitive to changes in cardiac preload and not to changes in heart rate (pacing) or afterload (phenylephrine).

What are then the mechanisms behind the fall in cardiac preload as a response to anaesthesia combined with PPV? A propofol-induced hypotension has been ascribed to reductions in preload and afterload by direct dilation of venous capacitance vessels [23, 24] and systemic resistance vessels [23–26] and decreased sympathetic activity [27, 28]. Moderate doses of remifentanil doesn’t seem to affect systemic capacitance vessels as it does not reduce cardiac filling pressures, stroke volume or cardiac output [29, 30]. Remifentanil has been shown to decrease heart rate and systemic vascular resistance [29, 30]. In the present study, propofol/remifentanil anaesthesia affected neither systemic vascular resistance nor arterial elastance, a measure of LV afterload, suggesting that the fall in cardiac index and MAP could to some extent be explained by a propofol/remifentanil-induced dilation of venous capacitance vessels, causing a preload decrease, together with a heart rate decrease.

In addition to the effects of propofol on LV GLS and RV free wall strain, one should also consider the effects of PPV on cardiac filling and thereby strain. During anaesthesia and in critically ill patients, PPV increases intrathoracic pressure, which will severely affect venous return and cardiac output [14]. It has been shown that the application of PPV plus positive end-expiratory pressure (PEEP) in mechanically ventilated patients decreases intra-thoracic blood volume [31] and LV and RV end-diastolic volumes as assessed by conventional echocardiohgraphy [32–35]. It is therefore likely that, at least to some extent, the lower LVEDVI and RVEDAI seen during anaesthesia and PPV, in the present study, were explained by PPV with the application of 5 cmH_2_O PEEP, which will decrease not only preload but also LV GLS and RV free wall strain. Franchi et al. investigated the effects of mechanical ventilation with PEEP on speckle tracking-derived myocardial strain in ICU patients under a multimodal ICU treatment and shown that increasing levels of PEEP causes a decrease in RV strain [36].

The fall in RV free wall strain and LV GLS induced by anaesthesia and PPV could to some extent be explained by a propofol-induced negative inotropic effect. Experimental data have provided evidence that propofol impairs myocardial contractility [37–41],while clinical data are somewhat controversial. Thus, Lepage et al. studied the effects of propofol on LV function by the use of radionuclide ventriculography and found that propofol induced a fall in cardiac output, stroke volume and cardiac filling pressures with no effects on LVEF or systemic vascular resistance, and therefore concluded that the propofol-induced decrease in cardiac output was caused by a fall in preload and not in impaired myocardial performance [42]. On the other hand, studies in patients on the effects of propofol on the end-systolic pressure-volume relationship have demonstrated that propofol impairs myocardial contractility [43, 44] In the present study, we measured LV end-systolic elastance non-invasively according to the so-called single beat method [17] and found that propofol impaired myocardial contractility. This method has been shown to have a good agreement with invasively measured LV end-systolic elastance [17].

One limitation of the present study is that we cannot distinguish the effects of the anaesthetics themselves to those of PPV on RV free wall strain and LV GLS, in the present study, as the patients need to be intubated and mechanically ventilated within minutes after induction of anaesthesia. Furthermore, the use of single bolus doses of ephedrine and phenylephrine in three patients could have attenuated the fall in blood pressure, cardiac filling and myocardial contractility. The strength is that we provide, for the first time, data on the effects of the transition from spontaneous breathing to anaesthesia combined with positive pressure breathing on LV and RV systolic function, as assessed by speckle tracking-derived myocardial strain of the LV and RV.

## Conclusion

General anesthesia combined with PPV reduces LV global longitudinal and RV free wall strain in patients with no heart disease. The fall in myocardial strain was most likely caused by a decrease in ventricular preload, caused by a propofol-induced dilation of venous capacitance vessels and PPV, together with a negative inotropic effect of propofol. These effects should be taken into account when evaluating heart function in surgical or critically ill patients subjected to anaesthesia /sedation and PPV.

### Ethical protocol

Protocol no. 477–17, approved by the Regional Ethical Review Board in Gothenburg: July 27th 2017 (www.epn.se).

## Data Availability

The datasets used and/or analyzed during the current study are available from the corresponding author on reasonable request.

## Abbreviations

A-max: Maximum flow velocity during late diastolic LV filling
ASA: American society of Anesthesiologists
BIS: Bispectral index
BSA: Body surface area
Ch: Chamber
Ch: Chamber
DAP: Diastolic arterial pressure
E-max: Maximum flow velocity during early diastolic LV filling
ICU: Intensive care unit
IVRT: Isovolumetric relaxation time
LV EF: Left ventricular ejection fraction
LV GLS: Left ventricular global longitudinal strain
LV: Left ventricular
LVEDVI: Left ventricular end-diastolic volume index
MAP: Mean arterial pressure
MAP: Mean arterial pressure
PEEP: Positive end-expiratory pressure
PPV: Positive pressure ventilation
RV: Right ventricular
RVEDAI: Right ventricular end-diastolic area index
SAP: systolic arterial pressure
SV: Stroke volume
SVI: Stroke volume index
SVRI: Systemic vascular resistance index
TVI-LVOT: Time velocity integral of the LV outflow tract

## References

[1] Amundsen BH, Helle-Valle T, Edvardsen T (2006). Noninvasive myocardial strain measurement by speckle tracking echocardiography: validation against sonomicrometry and tagged magnetic resonance imaging. J Am Coll Cardiol.

[2] Langeland S, Wouters PF, Claus P (2006). Experimental assessment of a new research tool for the estimation of two-dimensional myocardial strain. Ultrasound Med Biol.

[3] Mor-Avi V, Lang RM, Badano LP (2011). Current and evolving echocardiographic techniques for the quantitative evaluation of cardiac mechanics: ASE/EAE consensus statement on methodology and indications: endorsed by the Japanese Society of Echocardiography. J Am Soc Echocardiogr.

[4] Buckberg G, Hoffman JI, Mahajan A, Saleh S, Coghlan C (2008). Cardiac mechanics revisited: the relationship of cardiac architecture to ventricular function. Circulation.

[5] Farsalinos KE, Daraban AM, Unlu S, Thomas JD, Badano LP, Voigt JU (2015). Head-to-head comparison of global longitudinal strain measurements among nine different vendors: the EACVI/ASE inter-vendor comparison study. J Am Soc Echocardiogr.

[6] Urheim S, Edvardsen T, Torp H, Angelsen B, Smiseth OA (2000). Myocardial strain by Doppler echocardiography. Validation of a new method to quantify regional myocardial function. Circ.

[7] Rosner A, Bijnens B, Hansen M (2009). Left ventricular size determines tissue Doppler-derived longitudinal strain and strain rate. Eur J Echocardiogr.

[8] Ferferieva V, Van den Bergh A, Claus P (2012). The relative value of strain and strain rate for defining intrinsic myocardial function. Am J Physiol Heart Circ Physiol.

[9] Dahle GO, Stangeland L, Moen CA (2016). The influence of acute unloading on left ventricular strain and strain rate by speckle tracking echocardiography in a porcine model. Am J Physiol Heart Circ Physiol.

[10] Andersen NH, Terkelsen CJ, Sloth E, Poulsen SH (2004). Influence of preload alterations on parameters of systolic left ventricular long-axis function: a Doppler tissue study. J Am Soc Echocardiogr.

[11] Abali G, Tokgozoglu L, Ozcebe OI, Aytemir K, Nazli N (2005). Which Doppler parameters are load independent? A study in normal volunteers after blood donation. J Am Soc Echocardiogr.

[12] Mendes L, Ribeiras R, Adragao T (2008). Load-independent parameters of diastolic and systolic function by speckle tracking and tissue doppler in hemodialysis patients. Rev Port Cardiol.

[13] Burns A. T., La Gerche A., D'hooge J., MacIsaac A. I., Prior D. L. (2009). Left ventricular strain and strain rate: characterization of the effect of load in human subjects. European Journal of Echocardiography.

[14] Berger D, Takala J (2018). Determinants of systemic venous return and the impact of positive pressure ventilation. Ann Transl Med.

[15] Orde SR, Pulido JN, Masaki M (2014). Outcome prediction in sepsis: speckle tracking echocardiography based assessment of myocardial function. Crit Care.

[16] Dalla Keti, Bech-Hanssen Odd, Oras Jonatan, Naredi Silvana, Ricksten Sven-Erik (2018). Speckle tracking-vs conventional echocardiography for the detection of myocardial injury-A study on patients with subarachnoid haemorrhage. Acta Anaesthesiologica Scandinavica.

[17] Chen CH, Fetics B, Nevo E (2001). Noninvasive single-beat determination of left ventricular end-systolic elastance in humans. J Am Coll Cardiol.

[18] Vallabhajosyula S, Kumar M, Pandompatam G (2017). Prognostic impact of isolated right ventricular dysfunction in sepsis and septic shock: an 8-year historical cohort study. Ann Intensive Care.

[19] Fine NM, Chen L, Bastiansen PM (2015). Reference values for right ventricular strain in patients without cardiopulmonary disease: a prospective evaluation and meta-analysis. Echocardiography.

[20] Kissin I (2000). Depth of anesthesia and bispectral index monitoring. Anesth Analg.

[21] Sato M, Tanaka M, Umehara S, Nishikawa T (2005). Baroreflex control of heart rate during and after propofol infusion in humans. Br J Anaesth.

[22] Fredholm M, Jorgensen K, Houltz E, Ricksten SE (2017). Load-dependence of myocardial deformation variables - a clinical strain-echocardiographic study. Acta Anaesthesiol Scand.

[23] Bentley GN, Gent JP, Goodchild CS (1989). Vascular effects of propofol: smooth muscle relaxation in isolated veins and arteries. J Pharm Pharmacol.

[24] Rouby JJ, Andreev A, Leger P (1991). Peripheral vascular effects of thiopental and propofol in humans with artificial hearts. Anesthesiology.

[25] Patrick MR, Blair IJ, Feneck RO, Sebel PS (1985). A comparison of the haemodynamic effects of propofol (‘Diprivan’) and thiopentone in patients with coronary artery disease. Postgrad Med J.

[26] Boer F, Ros P, Bovill JG, van Brummelen P, van der Krogt J (1990). Effect of propofol on peripheral vascular resistance during cardiopulmonary bypass. Br J Anaesth.

[27] Sellgren J, Ejnell H, Elam M, Ponten J, Wallin BG (1994). Sympathetic muscle nerve activity, peripheral blood flows, and baroreceptor reflexes in humans during propofol anesthesia and surgery. Anesthesiology.

[28] Ebert TJ, Muzi M, Berens R, Goff D, Kampine JP (1992). Sympathetic responses to induction of anesthesia in humans with propofol or etomidate. Anesthesiology.

[29] Joo HS, Salasidis GC, Kataoka MT (2004). Comparison of bolus remifentanil versus bolus fentanyl for induction of anesthesia and tracheal intubation in patients with cardiac disease. J Cardiothorac Vasc Anesth.

[30] Yun SH, Kim JH, Kim HJ (2015). Comparison of the hemodynamic effects of nitroprusside and remifentanil for controlled hypotension during endoscopic sinus surgery. J Anesth.

[31] Brienza N, Dambrosio M, Cinnella G, Conte M, Puntillo N, Bruno F (1996). Effects of PEEP on intrathoracic and extrathoracic blood volumes evaluated with the COLD system in patients with acute respiratory failure. Preliminary study. Minerva Anestesiol.

[32] Terai C, Uenishi M, Sugimoto H, Shimazu T, Yoshioka T, Sugimoto T (1985). Transesophageal echocardiographic dimensional analysis of four cardiac chambers during positive end-expiratory pressure. Anesthesiology.

[33] Koolen JJ, Visser CA, Wever E, van Wezel H, Meyne NG, Dunning AJ (1987). Transesophageal two-dimensional echocardiographic evaluation of biventricular dimension and function during positive end-expiratory pressure ventilation after coronary artery bypass grafting. Am J Cardiol.

[34] Mitaka C, Nagura T, Sakanishi N, Tsunoda Y, Amaha K (1989). Two-dimensional echocardiographic evaluation of inferior vena cava, right ventricle, and left ventricle during positive-pressure ventilation with varying levels of positive end-expiratory pressure. Crit Care Med.

[35] Huemer G, Kolev N, Kurz A, Zimpfer M (1994). Influence of positive end-expiratory pressure on right and left ventricular performance assessed by Doppler two-dimensional echocardiography. Chest.

[36] Franchi F, Faltoni A, Cameli M, et al. Influence of positive end-expiratory pressure on myocardial strain assessed by speckle tracking echocardiography in mechanically ventilated patients. Biomed Res Int. 2013:918548. 10.1155/2013/918548 PMC3771268 24066303

[37] Zhou W, Fontenot HJ, Liu S, Kennedy RH (1997). Modulation of cardiac calcium channels by propofol. Anesthesiology.

[38] Coetzee A, Fourie P, Coetzee J (1989). Effect of various propofol plasma concentrations on regional myocardial contractility and left ventricular afterload. Anesth Analg.

[39] Brussel T, Theissen JL, Vigfusson G, Lunkenheimer PP, Van Aken H, Lawin P (1989). Hemodynamic and cardiodynamic effects of propofol and etomidate: negative inotropic properties of propofol. Anesth Analg.

[40] Sprung J, Ogletree-Hughes ML, McConnell BK, Zakhary DR, Smolsky SM, Moravec CS (2001). The effects of propofol on the contractility of failing and nonfailing human heart muscles. Anesth Analg.

[41] De Hert SG, Vermeyen KM, Adriaensen HF (1990). Influence of thiopental, etomidate, and propofol on regional myocardial function in the normal and acute ischemic heart segment in dogs. Anesth Analg.

[42] Lepage JY, Pinaud ML, Helias JH (1988). Left ventricular function during propofol and fentanyl anesthesia in patients with coronary artery disease: assessment with a radionuclide approach. Anesth Analg.

[43] Mulier JP, Wouters PF, Van Aken H, Vermaut G, Vandermeersch E (1991). Cardiodynamic effects of propofol in comparison with thiopental: assessment with a transesophageal echocardiographic approach. Anesth Analg.

[44] Martin C, Perrin G, Saux P, Papazian L, Albanese J, Gouin F (1994). Right ventricular end-systolic pressure-volume relation during propofol infusion. Acta Anaesthesiol Scand.

